# Vitamin D binding protein is not affected by high-dose vitamin D supplementation: a post hoc analysis of a randomised, placebo-controlled study

**DOI:** 10.1186/s13104-018-3725-7

**Published:** 2018-08-29

**Authors:** Linda Björkhem-Bergman, Emelie Torefalk, Lena Ekström, Peter Bergman

**Affiliations:** 1 Division of Clinical Geriatrics, Department of Neurobiology, Care Sciences and Society (NVS), Karolinska Institutet, Blickagången 16, Neo Floor 7, 141 83 Huddinge, Sweden; 2 ASIH Stockholm Södra, Långbro Park, Palliative Home Care and Hospice Ward, Bergtallsvägen 12, 125 59 Älvsjö, Sweden; 3 Division of Clinical Microbiology, Department of Laboratory Medicine, Karolinska Institutet, F68, 141 86 Stockholm, Sweden; 4 Division of Clinical Pharmacology, Department of Laboratory Medicine, Karolinska Institutet, C1-68, 141 86 Stockholm, Sweden

**Keywords:** Vitamin D, MRSA, Vitamin D binding protein, Free 25-hydroxyvitamin D

## Abstract

**Objectives:**

Vitamin D binding protein (VDBP) is the main transporter of 25-hydroxyvitamin D_3_ (25-OHD) in the circulation. The aim of this study was to investigate if VDBP is affected by high dose vitamin D supplementation and if VDBP-levels correlate with free 25-OHD. Correlation between free 25-OHD measured with ELISA and total 25-OHD in the circulation was also analysed. Plasma samples from a randomized, controlled trial in which persistent MRSA-carriers were randomized to treatment with vitamin D, 4000 IE/day, (n = 27) or placebo (n = 32) for 12 months were used. Plasma from baseline and after 6 months of treatment were analysed for VDBP, 25-OHD and free 25-OHD.

**Results:**

VDBP levels were not affected by vitamin D treatment, although the 25-OHD levels increased significantly in the vitamin D treated subjects. There was a strong correlation between 25-OHD and free 25-OHD (r^2^ = 0.68, p < 0.0001), while there was no correlation between VDBP and free 25-OHD. Thus, our data shows that VDBP are not affected by vitamin D supplementation and the levels of VDBP are not associated with the free fraction of 25-OHD. Since there was a strong correlation between free 25-OHD and total 25-OHD it appears to be sufficient to measure only total 25-OHD.

*Trial registration* http://www.clinicaltrials.gov; NCT02178488. Date of registration: June 30, 2014; Date of enrolment of the first participant: Dec 1, 2014

## Introduction

25-Hydroxyvitamin D_3_ (25-OHD) is considered as the best marker of vitamin D status in the human body [1]. Nearly all circulating 25-OHD are bound to proteins and it is only the limited fraction of free 25-OHD that has biological effects in target cells after conversion to the active 1,25-dihydroxyvitamin D. Vitamin D binding protein (VDBP), is the main transporter for 25-OHD in the circulation, transporting approximately 85% of total 25-OHD. A smaller proportion of 25-OHD, 10–15%, is transported by albumin [2]. VDBP, also known as GC-globulin, is synthesized in the liver and has numerous physiological roles, including immune-modulation, binding of fatty acids and regulation of bone development [3]. Thus, VDBP has many other functions than being a transporter and less than 5% of the binding sites on VDBP are occupied by 25-OHD [3].

Given that only the free 25-OHD is biologically active, it has been suggested that the levels of VDBP would determine the effects of vitamin D in the human body by regulating the levels of the free fraction of 25-OHD [4]. This hypothesis was the basis for a highly cited study that demonstrated that Afro-Americans had lower levels of VDBP—but similar levels of free 25OHD—than Caucasians [5]. However, other studies have failed to show ethnical differences in VDBP-levels [6]. The discrepancy between various studies was recently explained and found to be caused by the use of different kits for VDBP-analysis. In fact, VDBP-kits that used a monoclonal antibody failed to detect different variants of the highly polymorphic VDBP-protein, which could be detected by ELISA-kits using polyclonal sera [7].

Previously, free 25-OHD was determined by the use of a formula based on 25-OHD, VDBP and albumin-levels [8]. Recently, a commercially available ELISA was developed to measure the free fraction of 25-OHD in plasma directly, which has replaced the indirect calculation method [9].

We had access to samples from a previous study where vitamin D was given to eradicate carriage of methicillin resistant *Staphylococcus aureus* (MRSA). The rationale behind this study was that (i) vitamin D induces antimicrobial peptides with anti-staphylococcal activity; (ii) MRSA-carriers have lower levels of 25OHD than non-carriers and (iii) vitamin D supplementation previously has been shown to reduce carriage rates of *S. aureus*. Vitamin D supplementation did not affect carriage rate of MRSA in this study [10]. However, the role of VDBP and free 25-OHD in the context of a vitamin D supplementation study has not been well described before. Thus, using these samples, we set out to study how VDBP-levels were affected by vitamin D supplementation. We also investigated whether there was a correlation between free 25-OHD and VDBP in plasma as well as between free 25-OHD and total 25-OHD.

## Main text

### Method

The D-STAPH study was a double blind, randomised and placebo-controlled trial performed during 2014–2015 [10]. The aim of the study was to investigate if vitamin D treatment could eradicate methicillin resistant *S. aureus* (MRSA) in persistent MRSA-carriers. Persistent MRSA-carriers were given the study drug (4000 IU vitamin D or placebo daily) during a 12 months period and were followed closely during this period with visits every 3 months. No effects of vitamin D supplementation on MRSA-carriage could be observed in this trial [10].

Plasma samples from baseline and 6 months were analysed in the current project. There were samples available from 27 vitamin D treated subjects and 32 placebo treated subjects. Measurements of total 25-OHD were made with chemiluminescence immunoassay (CLIA) on a LIAISON-instrument (DiaSorin Inc, Stillwater, MN, USA,) with a detectable range of 7.5–175 nmol/L, CV 2–5% at the Department of Clinical Chemistry, Karolinska University Hospital, Stockholm, Sweden. Vitamin D binding protein was measured with ELISA, using polyclonal anti-VDBP antibodies according to the manufacturer’s protocol (Immundiagnostik AG, Bensheim, Germany). Free 25-OHD was measured with quantitative two-step ELISA immunoassay (Future Diagnostics, DiaSource, Wijc, The Netherlands) [9].

### Statistical analysis

Statistical analyses were performed using Graph Pad Prism vs 6.0. When comparing 25-OHD levels between the different groups, student’s t-test was used since the data showed Gaussian distribution. Correlation between 25-OHD levels and free 25-OHD and between free 25-OHD and VDBP levels was determined with linear regression.

### Results

**Fig. 1:**
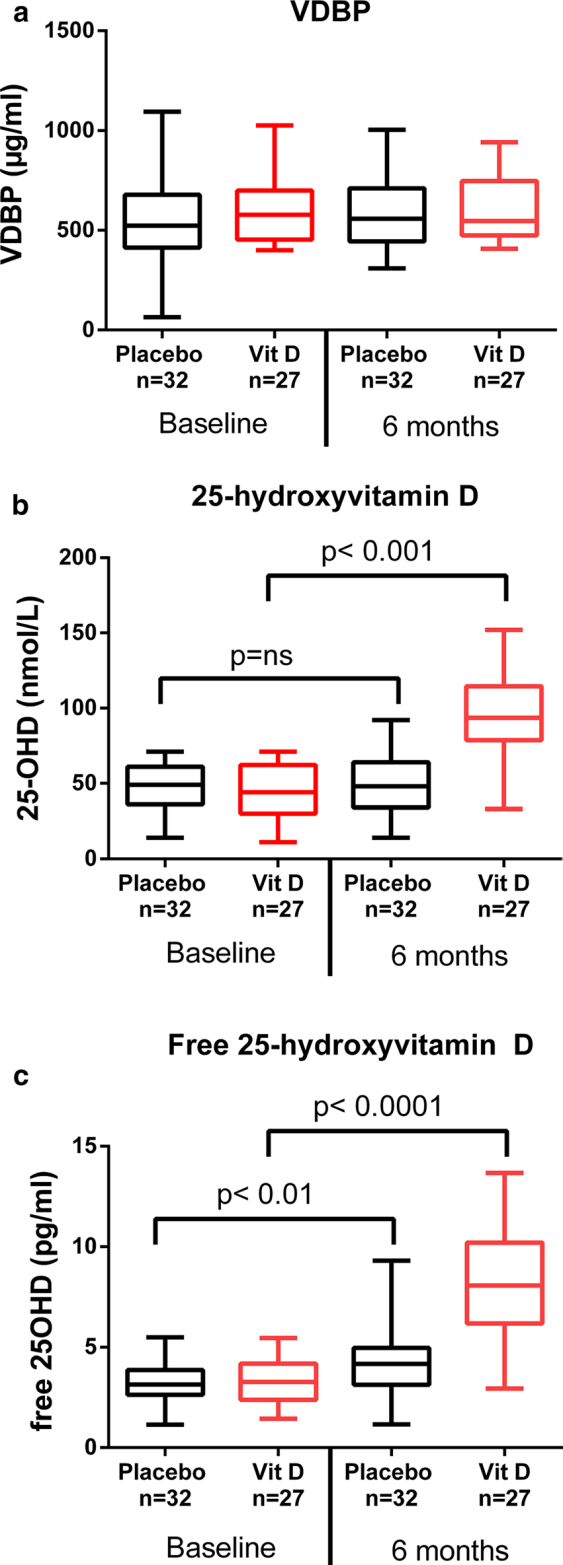
Plasma levels of **a** vitamin D binding protein (VDBP), **b** 25-hydroxyvitamin D (25-OHD) and **c** free fraction of 25-OHD in the placebo group and the vitamin D treated group at baseline and after 6 months in the original study [10]

The levels of VDBP were similar at baseline in both groups and were not affected over time or by high dose vitamin D supplementation for 6 months (Fig. 1a). Vitamin D supplementation resulted in significantly increased levels of 25-OHD as expected, p < 0.001 (Fig. 1b), while the levels in the placebo-group did not increase significantly. Free 25-OHD increased in a similar way in the vitamin D group (Fig. 1c). However, also in the placebo group a slight, but statistically significant, increase of free 25-OHD was observed (Fig. 1c).

**Fig. 2:**
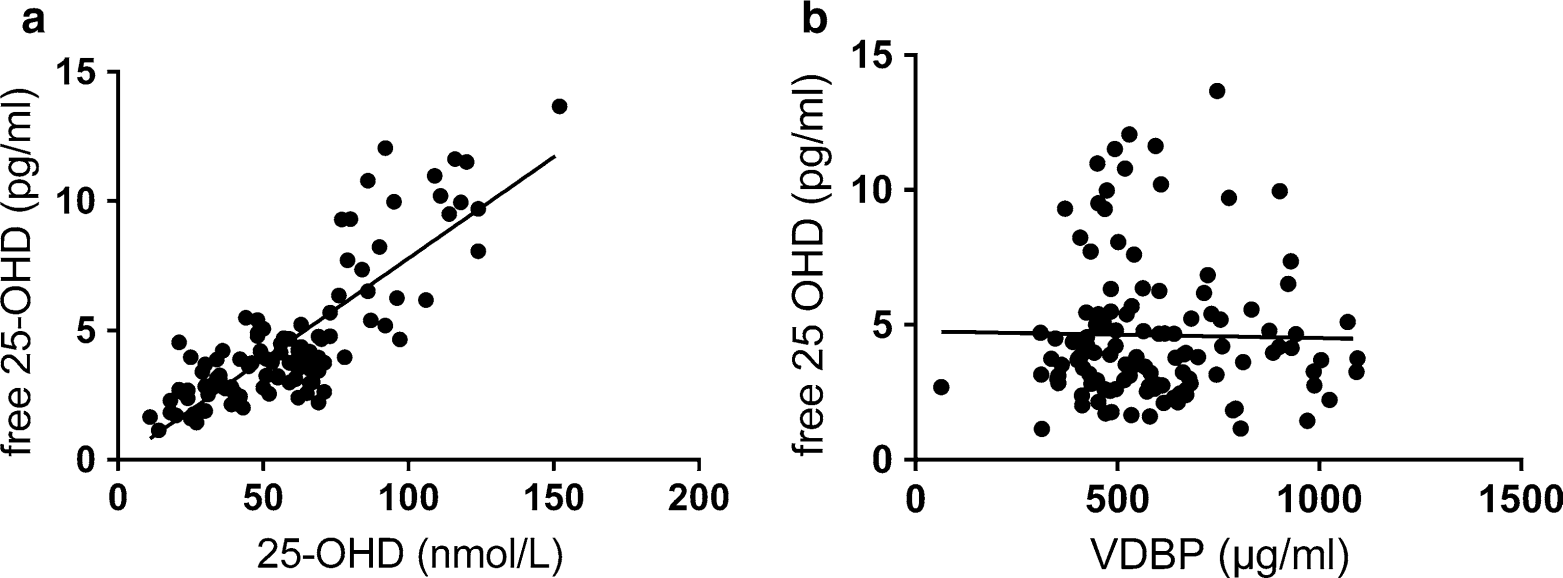
Correlation between **a** free 25-hydroxyvitamin D (25-OHD) and **b** 25-OHD and between free 25-OHD and vitamin D binding protein (VDBP) in plasma samples from the original study at baseline and after 6 months (n = 118)

There was a strong correlation between 25-OHD levels and free 25-OHD, linear regression showed r^2^ = 0.68, p < 0.0001 (Fig. 2a). In contrast, no significant correlation between free 25-OHD and VDBP levels was observed (Fig. 2b).

There was no significant correlation between the ratio free 25-OHD/total 25-OHD and VDBP, linear regression showed r^2^ = 0.01, p = 0.31 (data not shown).

There was no correlation between VDBP levels and MRSA-carriage after 6 months (data not shown). Albumin-levels at baseline were within the normal range 33–48 g/L, mean 37 g/L, median 39 g/L, and did not change during the study.

### Discussion

Here we show that VDBP-levels in plasma are not affected by high-dose vitamin D supplementation for 6 months. In addition, our findings suggest that VDBP-levels do not correlate with the levels of free 25-OHD in the circulation. This is in contrast to some previous reports also using direct methods to measure the free fraction [11–13]. However, taking into account that less than 5% of the binding sites on VDBP are occupied by 25-OHD [3], it is reasonable to suggest that VDBP levels only have a minor impact on the levels of free 25-OHD. Notably, albumin-levels were within the normal range in all participants and remained unchanged during the study. Unexpectedly, the levels of free vitamin D showed a slight increase in the placebo-group. At this point, we have no explanation for this observation.

Since there was a strong correlation between 25-OHD levels and free 25-OHD, it may be sufficient to measure only total 25-OHD in order to obtain information on the vitamin D status of individual patients in vitamin D supplementation studies.

However, it should be noted that the patients in this study were generally healthy and recruited based on their status as persistent MRSA-carriers. In an early study on patients with liver disease, it was shown that the ratio between free and total vitamin D correlated with VDBP-levels or albumin, whereas there was no correlation between the specific levels of free or total vitamin D with VDBP or albumin [11]. Therefore, there could still be a role for analyses of free 25-OHD in vitamin D supplementation trials, especially in populations with altered VDBP-levels, such as cirrhotic, obese and pregnant individuals [14]. Today, there are accurate and simple ELISA methods available on the market for analyses of free 25-OHD.

A recent cross-sectional study assessed free 25-OHD in a large cohort and found that cirrhotic patients had lower VDBP-levels and higher fraction of free 25-OHD-levels. Reciprocally, pregnant women had higher VDBP-levels but—somewhat unexpected—normal levels of free 25-OHD [14]. Combined, these recent results suggest that the relations between VDBP, total 25-OHD and free 25-OHD are complex and that further studies are needed to determine the role of free 25-OHD analyses in health and disease.

## Limitations

The most important limitation is the relatively small study cohort (vitamin D n = 27; placebo n = 32), which could have hidden minor associations in the material. However, if there was a true linear correlation between VDBP and free 25-OHD, as suggested by other researchers, we would have expected at least a trend towards correlation, but this was not the case. As expected, there was a strong correlation between total 25-OHD and free 25-OHD. A final limitation was the lack of information of different VDBP haplotypes, which could have affected the results. However, since a polyclonal antibody was used in the ELISA assay the risk for this potential problem is limited.

## Abbreviations

25-OHD: 25-hydroxyvitamin D
MRSA: methicillin resistant *Staphylococcus aureus*
VDBP: vitamin D binding protein
